# Supplementary material for: Electrical addressing of exceptional points in compact plasmonic structures
Source: Nanophotonics. 2023 Apr 19;12(11):2029–39. doi: 10.1515/nanoph-2023-0125 (PMC11501537; doi:10.1515/nanoph-2023-0125)
Supplement: Supplementary file 3 — Supplementary Material Details [file j_nanoph-2023-0125_suppl_001.pdf]

Supplementary Material

Electrical addressing of exceptional points in compact plasmonic structures

Hoon Yeub Jeong[1], Yeonsoo Lim[1], Jungho Han[1], Soo-Chan An[1], and Young Chul Jun[1,2]

[1]Department of Materials Science and Engineering, Ulsan National Institute of Science and Technology (UNIST), Ulsan 44919, Republic of Korea

[2]Graduate School of Semiconductor Materials and Devices Engineering, UNIST, Ulsan 44919, Republic of Korea

- **Figure S1:** Snapshot of the time-domain simulation
- **Figure S2:** Simulated complex eigenfrequencies for different metal-line angles.
- **Figure S3:** Simulated eigenmode profile away from the EP condition
- **Figure S4:** Simulated eigenmode profile near the EP condition
- **Figure S5:** Mode-splitting simulations near the EP
- **Figure S6:** Experimental reflection spectra together with fitting curves
- **Figure S7:** Simulated reflection spectrum together with a fitting curve
- **Figure S8:** Experimentally measured phase spectra

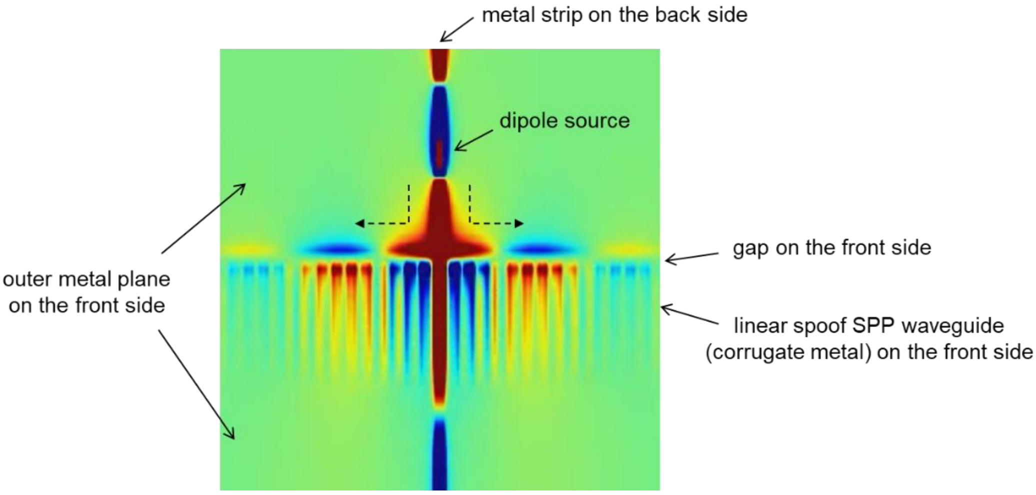

Figure S1. Snapshot of the time-domain simulation (Lumerical FDTD). The metal line on the backside is aligned to pass the middle of a one-dimensional (straight) spoof plasmon waveguide. A propagating wave along the metal line is directly excited by a point dipole source. The metal line works as a splitter (see Supplementary Video V1). Incident waves are split into the two opposite directions of the spoof plasmon waveguide. In the case of our circular localized spoof plasmon (LSP) resonator, the split waves become clockwise and counterclockwise circulating plasmon waves. Their interactions form different modes depending on the metal-line angle.

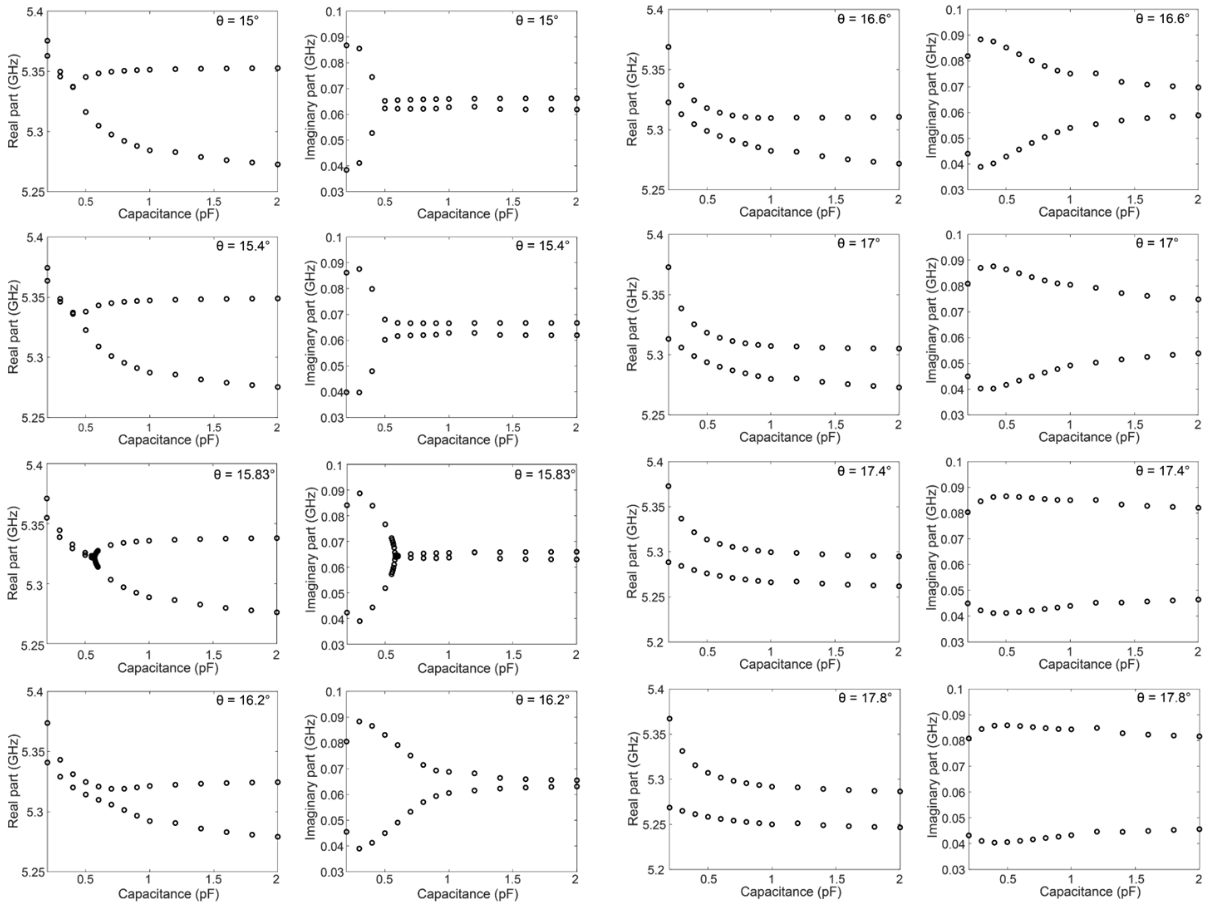

Figure S2. Simulated complex eigenfrequencies for different metal-line angles. Real and Imaginary parts of the complex eigenfrequencies are shown for θ = 15° ~ 17.8°. Between θ = 15° and 16°, both real and imaginary parts of the two eigenmodes become very close. The real and imaginary parts of the complex eigenfrequencies of the two eigenmodes nearly coalesce simultaneously at θ = 15.83°, corresponding to an exceptional point (EP).

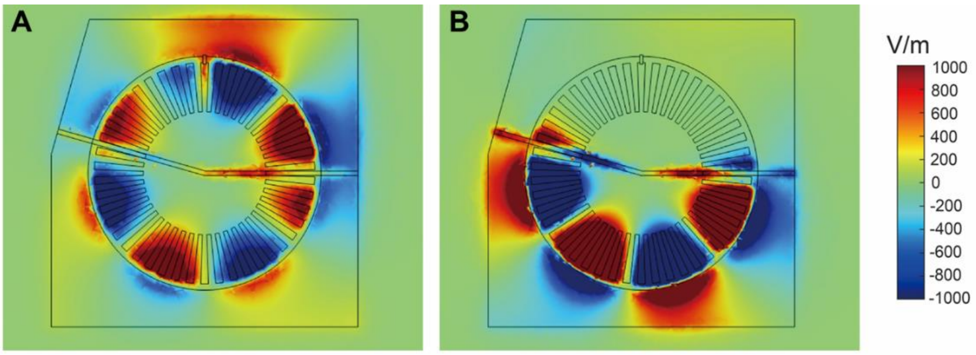

Figure S3. Simulated eigenmode profile (E_z) at $\theta = 15.83°$ and capacitance of 2 pF (away from the EP condition). (A) and (B) show the field profiles at 5.27 GHz and 5.34 GHz, respectively. A cutting corner was introduced at the upper-left corner for the angled metal line.

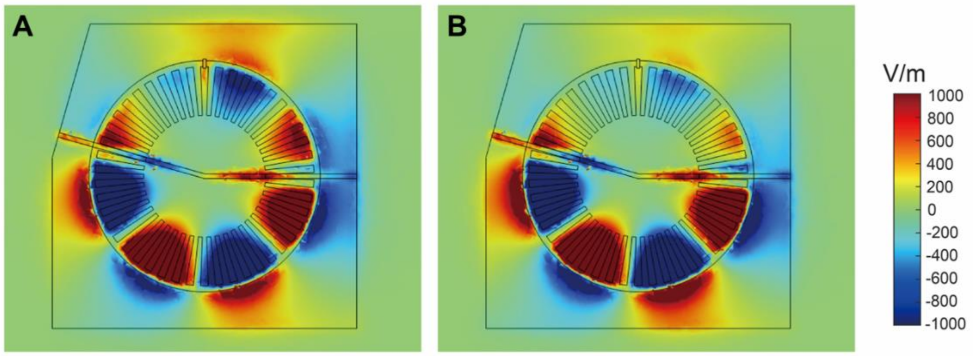

Figure S4. Simulated eigenmode profile (E_z) at $\theta = 15.83°$ and capacitance of 0.585 pF (near the EP condition). (A) and (B) are the field profiles at 5.32 GHz. The field profiles of the two eigenmodes become similar.

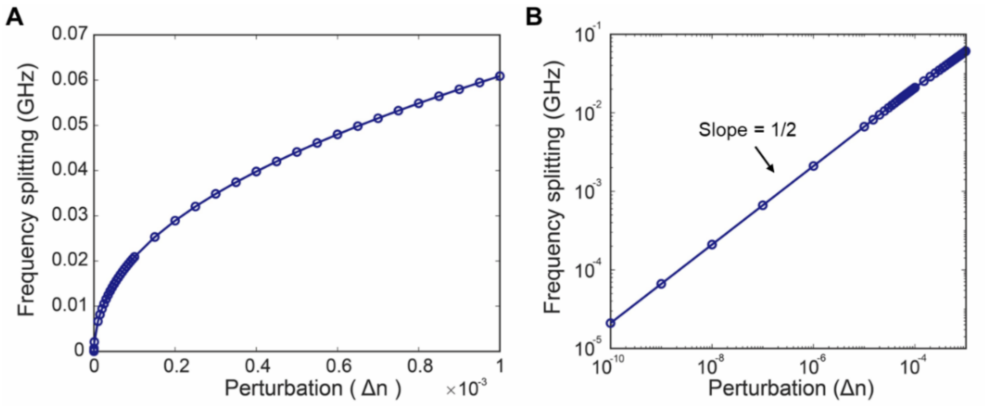

Figure S5. Mode-splitting simulations near the EP in our spoof plasmon structure. In the simulation, the refractive index of the surrounding medium is gradually increased (n = 1 + Δn), and changes in the mode splitting are measured. Fitting of this mode splitting in the log-log plot perfectly matches a square-root curve (i.e., slope = 1/2) under small perturbations. This is clearly different from other mode-splitting mechanisms (diabolic points) which exhibit linear dependence under perturbations.

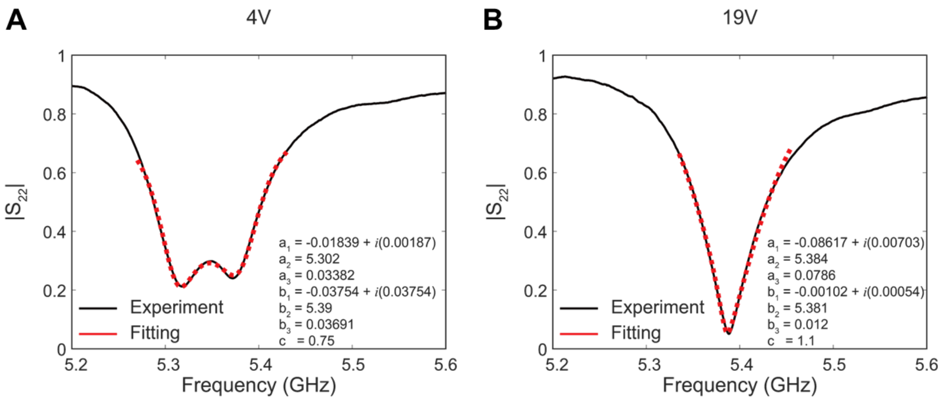

Figure S6. (A) and (B) Experimental reflection spectra at 4 V and 19 V, respectively, together with fitting curves (red dotted lines). The metal-line angle is θ = 16° in both cases. Fitting parameters are also indicated in each case. The number of fitting parameters was minimized in the initial fitting using proper simplifications. Then, fitting was repeated for all parameters to fine tune and optimize the fitting parameters.

(i) Figure S6A shows the experimental reflection spectrum at 4 V, where the reflection spectrum shows two, separate resonance dips. In this case, initial fitting was conducted separately for individual resonances. Then, the whole spectrum was fitted again for fine tuning.

(ii) Figure S6B shows the experimental reflection spectrum at 19 V (away from the EP condition), where the reflection spectrum shows a single (nearly overlapped) resonance dip. In this case, initial fitting was conducted using reasonable estimations for the resonance frequencies ($a_2 \approx b_2$) and the background level c. Then, fitting was repeated to optimize the whole fitting parameters.

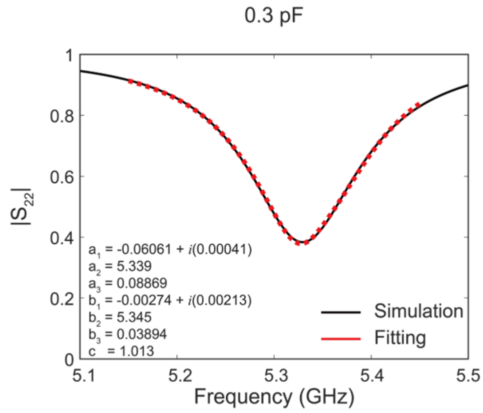

Figure S7. Simulated reflection spectrum at a capacitance of 0.3 pF together with a fitting curve (red dotted line) (θ = 15.83°). Fitting parameters are also indicated.

To confirm the experimental data fitting further, fitting is also conducted using the simulated reflection spectra. In this case, the real and imaginary parts of the complex resonance frequencies (a_2, a_3, b_2, b_3) are obtained from separate simulations (eigenfrequency simulations). Then, Eq. (4) was fitted to the simulated reflection spectrum to determine other parameters. Figure S7 shows an exemplary case at a capacitance of 0.3 pF (away from the EP condition) in Figure 5 (i.e., nearly overlapped resonance at θ = 15.83°). It is found that an overall trend in the fitting parameters of the simulation is very similar to that in the experimental data fitting. For example, for the nearly overlapped resonance, the high-Q mode ($|b_3| < |a_3|$) has a much smaller resonance amplitude ($|b_1/b_3| < |a_1/a_3|$) in both experiment and simulation (Figures S6B and S7). (However, we note that the experimental reflection spectrum has a sharper resonance and smaller b_3 than the simulated spectrum)

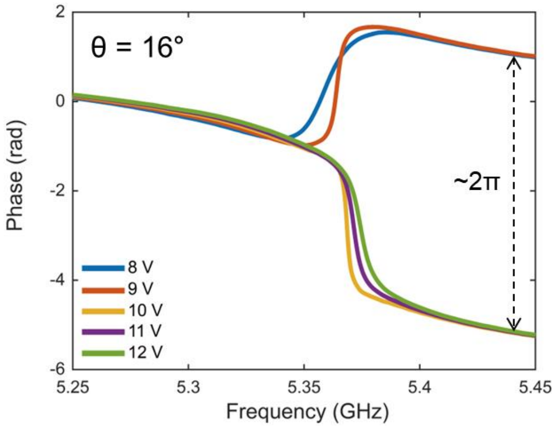

Figure S8. Experimentally measured phase spectra for several different voltages (θ = 16°). The phase spectra were also measured at Port 2. When the voltage changes from 9 to 10 V, an abrupt change in the phase spectrum occurs owing to the phase singularity.
